# *Chlamydia* exploit the mammalian tryptophan-depletion defense strategy as a counter-defensive cue to trigger a survival state of persistence

**DOI:** 10.3389/fcimb.2014.00017

**Published:** 2014-02-28

**Authors:** Carol A. Bonner, Gerald I. Byrne, Roy A. Jensen

**Affiliations:** ^1^Microbiology and Cell Science, Emerson Hall, University of FloridaGainesville, FL, USA; ^2^Department of Microbiology, Immunology and Biochemistry, University of Tennessee Health Science CenterMemphis, TN, USA

**Keywords:** tryptophan, chlamydiae, persistence, menaquinone biosynthesis, Up-Trp selection, Down-Trp selection, reductive evolution

## Abstract

We previously proposed that in *Chlamydiaceae* rapid vegetative growth and a quiescent state of survival (persistence) depend upon alternative protein translational profiles dictated by host tryptophan (Trp) availability. These alternative profiles correspond, respectively, with a set of chlamydial proteins having higher-than-predicted contents of Trp (“Up-Trp” selection), or with another set exhibiting lower-than-predicted contents of Trp (“Down-Trp” selection). A comparative evaluation of *Chlamydiaceae* proteomes for Trp content has now been extended to a number of other taxon families within the *Chlamydiales* Order. At the Order level, elevated Trp content occurs for transporters of nucleotides, S-adenosylmethionine (SAM), dicarboxylate substrates, and Trp itself. For Trp and nucleotide transporters, this is even more pronounced in other chlamydiae families (*Parachlamydiaceae*, *Waddliaceae*, and *Simkaniaceae)* due to extensive paralog expansion. This suggests that intracellular Trp availability served as an ancient survival cue for enhancement or restraint of chlamydial metabolism in the common *Chlamydiales* ancestor. The *Chlamydiaceae* Family further strengthened Up-Trp selection for proteins that function in cell division, lipopolysaccharide biosynthesis, and methyltransferase reactions. Some proteins that exhibit Up-Trp selection are uniquely present in the *Chlamydiaceae*, e.g., cytotoxin and the paralog families of polymorphic membrane proteins (Pmp's). A striking instance of Down-Trp selection in the *Chlamydiaceae* is the chorismate biosynthesis pathway and the connecting menaquinone pathway. The newly recognized 1,4-dihydroxy-6-napthoate pathway of menaquinone biosynthesis operates in *Chlamydiaceae*, whereas the classic 2-napthoate pathway is used in the other *Chlamydiales* families. Because of the extreme Down-Trp selection, it would appear that menaquinone biosynthesis is particularly important to the integrity of the persistent state maintained under conditions of severe Trp limitation, and may thus be critical for perpetuation of chronic disease states.

## Introduction

### Persistence

One form of immune evasion is a developmental state of the *Chlamydiaceae* Family called “persistence” that is triggered as a response to stress stimuli that cue an impending immune response by the host (Beatty et al., 1994). Persistence is a sophisticated survival mode, whereby a state of reversible quiescence is implemented. Recent reviews have been published in which the nature of persistence has been discussed (Hogan et al., 2004; Wyrick, 2010; Schoborg, 2011). Beyond the general impact for pathogen survival, persistence can be equated with chronic disease states of the host, e.g., inflammatory arthritis in humans (Beatty et al., 1994; Hogan et al., 2004).

### Up-Trp and Down-Trp sets of proteins

A chlamydial mechanism has evolved which mutes the expression of gene products necessary for the rapid pathogen proliferation associated with acute disease, but which is permissive to the expression of gene products that underlie the unique morphological and developmental characteristics of persistence. This switch from one translational profile to an alternative translational profile was proposed by Lo et al. (2012) to be accomplished by maximizing the tryptophan (Trp) content (Up-Trp selection) of some key proteins needed to sustain rapid proliferation, e.g., ADP/ATP translocase, hexose-phosphate transporter, phosphoenolpyruvate (PEP) carboxykinase, the Trp transporter, the polymorphic membrane protein (Pmp) superfamily for cell adhesion and antigenic variation, and components of the cell-division pathway—at the same time minimizing the Trp content (Down-Trp selection) of other proteins needed to maintain the state of persistence. A bioinformatic analysis of the Trp content of the proteomes of six *Chlamydiaceae* genomes was carried out (Lo et al., 2012) in which the Trp content of each protein was expressed as a “p/P ratio”, i.e., (Trp content of a given protein); (Trp content of its Proteome). *Protochlamydia amoebophila* (Pamo) was included as a phylogenetically near out-group proteome and *E. coli* (Ecol) was used as a phylogenetically distant out-group proteome. Trp content in proteomes increases with increase in genomic G/C content. Thus, p/P ratios were used to normalize the Trp-content data in order to facilitate the comparison of different organisms.

### Rationale to explain feasibility of Up-Trp selection as a pathogen strategy

The biosynthesis of Trp is particularly costly because of the energy-metabolite resources needed, which makes it understandable why chlamydiae (and many other pathogens and symbionts) have evolved the luxury of reliance upon host resources for pre-formed Trp to conserve energy. Since Trp is thus defined as a metabolite of particular value, a reasonable question arises as to how a pathogen strategy of selectively increased Trp usage to accommodate the translational profile of proteins important for rapid pathogen propagation could be feasible. In part, this is explained by the offsetting effect of Down-Trp selection for the set of proteins that is important for maintenance of the persistent state. Significantly, Up-Trp selection is further facilitated with minimal overall Trp usage by a number of innovative tactics: (i) A regionally dense concentration of Trp-residue placements can block translation of a given protein having an overall Trp content that is average or even low, (ii) Amplification of the Trp content of a single “master” protein required for expression or maturation of multiple “slave” proteins means that the suite of slave proteins remain sensitive to the controlling influence of Trp depletion, even though their Trp content might be low, and (iii) An elevated Trp content of just one or a few component enzymes in complex, multi-step pathways can create an Achilles-heel vulnerability of the overall pathway.

### Phylogenetic extension of the Trp-content analysis to the *Chlamydiales* order

The *Chlamydiaceae* are well characterized obligate intracellular pathogens of humans and animals. In contrast, other families of the *Chlamydiales* Order have only recently come under the extensive scrutiny enabled by genome sequencing and bioinformatic analysis (Horn, 2008; Collingro et al., 2011). Three of these *Chlamydiae* families are the *Simkaniaceae*, *Parachlamydiaceae*, and *Waddliaceae*. The natural eukaryotic hosts for these families appear to be protozoans such as amoebae, although members of these chlamydial families exhibit a broad host range and have been associated with mammalian disease as emerging pathogens (Greub and Raoult, 2002). Phylogenetic trees indicate that of the four taxon families, the *Chlamydiaceae* branch at the deepest position (Collingro et al., 2011). This may seem surprising because their mammalian hosts, with which there are many co-evolved characteristics, appeared quite recently on the geologic timescale. It therefore seems likely that the *Chlamydiaceae* emerged recently as mammalian pathogens from an ancient, early-divergent lineage of the *Chlamydiales*, other descendents of which may yet be discovered in association with thus-far unknown hosts. The *Chlamydiaceae* possess the smallest, most evolutionarily reductive genome of the four taxon families, probably reflecting the niche specialization that is the relatively stable and homeostatic environment of the mammalian host. No free-living relatives have yet been described within the *Chlamydiales*. Thus far, all *Chlamydiales* have in common: (i) an obligate intracellular lifestyle as pathogens or endosymbionts, (ii) the targeting of eukaryotic organisms as hosts, and (iii) a similar developmental routine that transitions between infectious elementary bodies (EBs) and proliferative reticulate bodies (RBs). Since previous observations made with Pamo (Lo et al., 2012) had hinted that some events of Up-Trp and Down-Trp selection had occurred prior to the divergence of *Chlamydiaceae*, it was of interest to sort out which Up-Trp/Down-Trp selections were specific to the *Chlamydiaceae* and which exhibited a broader distribution among the *Chlamydiales*.

## Different mechanisms yield a common outcome of Trp depletion in man and mouse

The Trp starvation mechanism is best understood in the human/*Chlamydia trachomatis* host/pathogen relationship, but the similarity of Up-Trp and Down-Trp proteomic profiles in all pathogenic *Chlamydiaceae* implies that Trp availability is an underlying cue relied upon by this entire family of pathogens to trigger developmental transitions (Lo et al., 2012). However, the diversity of host organisms parasitized by the *Chlamydiaceae* deploy different immune-response tactics that do not necessarily implement the direct cytosolic degradation of Trp seen in *C. trachomatis*. How the same ultimate outcome of Trp depletion might have come to be is illustrated by a comparison of the scenarios of co-evolved features at work in the human/*C. trachomatis* and mouse/*C. muridarum* pairings of host and pathogen. Here replacement of an ancestral IFN-γ/GTPase/cytotoxin/Trp-depletion mechanism in the mammalian lineage by a contemporary IFN-γ/indoleamine dioxygenase/Trp-depletion mechanism in humans was proposed (Lo et al., 2012). The conclusion that the IFN-γ/GTPase/cytotoxin/Trp-depletion mechanism is the ancestral mechanism is the most parsimonious evolutionary possibility based upon the broadly distributed IFN-γ induced p47 GTPase/cytotoxin host/pathogen combination in mammals compared to the absence of the latter in primates which exhibit instead a phylogenetically narrow distribution of IFN-γ induced indoleamine dioxygenase (IDO).

### The mouse IFN-γ/GTPase/cytotoxin/Trp-depletion mechanism

**Figure 1:**
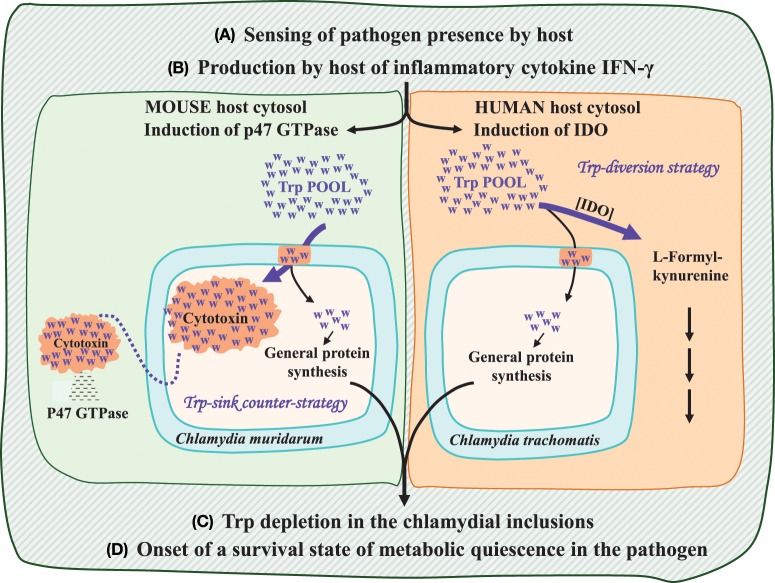
**Common and differential steps of the mammalian immune response: comparison of the mouse host (left side) with the human host (right side).** Common steps are listed as **(A–D)**. Progression from step **(B)** to step **(C)** is mediated by different mechanisms. The human host deploys a strategy of Trp depletion via the action of an IFN-γ induced indoleamine dioxygenase (IDO). In the mouse host p47 GTPase production is the anti-chlamydial strategy that, in turn, is met with the chlamydial counter-strategy of cytotoxin production and its subsequent export from the chlamydial inclusion body. The cytotoxin molecules can be viewed as a Trp sink, with the exportation step effectively accomplishing Trp depletion. Free Trp molecules are depicted as blue “w”s, whereas proteinaceous Trp molecules are shown against bright orange backgrounds.

As illustrated in Figure 1, four general steps are common to the generation of persistence in the two host/pathogen combinations. However, the specific events that intervene between steps (B) and (C) are quite different. The overall mechanism seen in mouse, in contrast with that of man, presumably resembles the mechanism present in the common ancestor of mammals. Here production of interferon-gamma (IFN-γ) induces p47 GTPase, which possesses membrane regulatory features that are effective against compartmented pathogens (Kim et al., 2011). The pathogen defense response, in turn, is to produce large, exportable cytotoxin molecules, virulence factors which target the p47 GTPase proteins (Bourne et al., 1990). The high-Trp cytotoxin molecules are very large (>3000 amino acids per monomer) and have been hypothesized (Lo et al., 2012) to act as Trp sinks within the inclusion, with cytotoxin export then completing the process creating a state of Trp depletion in the pathogen. The expenditure of Trp for cytotoxin translation is accentuated by the very large size of the protein and by its probable existence in a multimeric state (Voth et al., 2004). In the mouse pathogen, *C. muridarum*, flow of Trp to cytotoxin is at an even greater extreme because three paralog proteins are synthesized due to multiple gene duplications which generated three tandem paralog genes.

### The human IFN-γ/indoleamine dioxygenase/Trp-depletion mechanism

In man and other primates, IFN-γ manifests a quite different outcome, as visualized on the right side of Figure 1. Induction of the p47 GTPase family by IFN-γ does not occur in the primate lineage (Bekpen et al., 2005), instead being replaced by induction of IDO. The utilization of Trp as substrate by IDO directly creates a state of Trp depletion in the host cytosol. The ascension of IDO as a major player in immune surveillance in humans might be related to the increasing recognition that IDO induction and the consequent Trp depletion may be effective against some other intracellular pathogens, and even some extracellular pathogens. Indeed antiviral effects mediated by IDO have been reported as well (See Lo et al., 2012 and references therein). A very limited entry of Trp into the highly truncated fragments of the cytotoxin made by *C. trachomatis* is a consequence of the evolutionary disruption of the cytotoxin gene. Thus, the comparison given in Figure 1 illustrates how the different effect of IFN-γ mobilization (step B) in mouse and man can unfold to give the same Trp-depletion result (step C). This occurs via direct exclusion of Trp from the pathogen inclusion in the first place (man) or indirectly, by generation of exportable cytotoxin (an effective Trp sink) to combat p47 GTPase (mouse).

### Opposite adjustments of cytotoxin in *C. muridarum* and *C. trachomatis*

In the absence of homologs from other *Chlamydiales* families, cytotoxin is concluded to have undergone Up-Trp selection by comparison of p/P Trp ratios with those of distant homologs available elsewhere (see Lo et al., 2012 for detailed comparisons). In the case of cytotoxin, an ancestral state of high Trp content produced by Up-Trp selection has been subject to very recent, and quite opposite adjustments in two species. Thus, Up-Trp selection has been further increased to a dramatic extent in the mouse pathogen (*Chlamydia muridarum*) via several rounds of paralog expansion, but drastically negated in the human pathogen (*C. trachomatis*) via frameshift mutations.

## Crucial features of the Trp-responsive network supporting rapid pathogen proliferation

**Figure 2:**
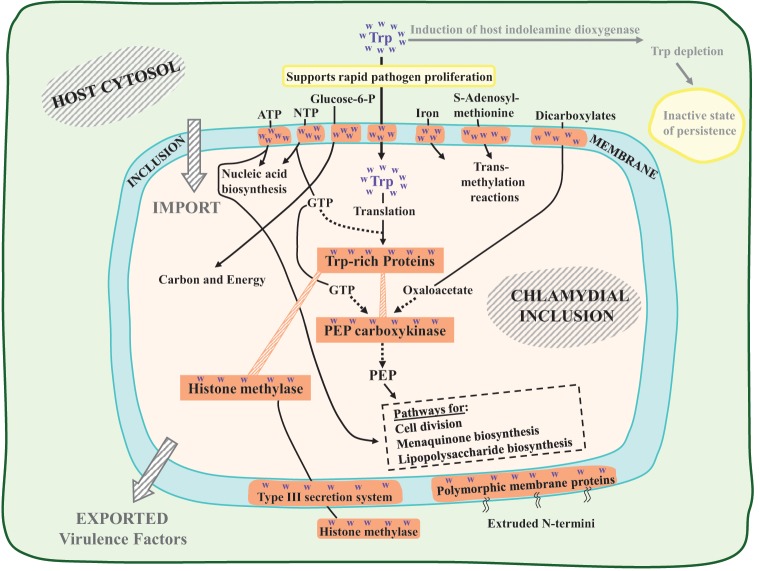
**Key elements in the *C. trachomatis* pathogen of a response network which is tuned to adequate Trp availability from human host cytosol.** Two alternative flow routes for cytosolic Trp are indicated. The rightward flow route (dimmed) is initiated with the induction of indoleamine dioxygenase (IDO). When Trp is fully available prior to the triggering of any process accomplishing Trp depletion (downward bold arrow), a schedule of rapid pathogen proliferation is supported by expression of key Trp-rich proteins. Many of these are membrane proteins that promote transport into the inclusion body of important metabolites derived from the host cytosol, illustrated by those shown across the top of the diagrammed inclusion membrane. The *C. trachomatis* PEP carboxykinase is positioned at the center of an interactive assemblage of Trp-rich proteins, as discussed extensively in the text. The export (or partial export) of virulence factors is illustrated by histone methylase and by the family of polymorphic membrane proteins (Pmp's).

Figure 2 depicts alternative fates of Trp molecules in the human host cytosol: (i) as substrate for IDO with the consequence of Trp depletion (rightward arrow), or (ii) transport into the pathogen inclusion via two TyrP-family permeases (downward arrow). Figure 2 is intended to display alternative (ii), i.e., the scenario that unfolds when immune surveillance has not yet been triggered to activate IDO catalysis. A selection of high-Trp proteins that are critical for rapid proliferation of *C. trachomatis* under conditions of acute disease is diagrammed in Figure 2. These include proteins that accomplish the import of essential nutrients from the host, proteins that accommodate the export of virulence factors that interact with the host biochemical network, and proteins that play key roles in basic metabolism.

### Permeases

Membrane proteins generally exploit Trp for its unique physical properties, and Up-Trp selection has further increased the Trp content of a number of permeases. Noteworthy transporters of high Trp content shown across the top of Figure 2 include a narrow-specificity ATP transporter, a broad-specificity nucleotide triphosphate (NTP) transporter, and transporters for glucose-6-phosphate, iron, S-adenosylmethionine (SAM), and dicarboxylate keto acids. Centrally, the import of Trp itself is mediated in *Chlamydiaceae* by one or two transporters of the TyrP family that have high-Trp content. Thus, import of Trp is self-limiting in the sense that any decrease of TyrP synthesis during starvation for Trp will tend to abort the entire pyramid of high-Trp proteins that depend upon TyrP for import of a Trp supply.

### Virulence factors

Histone methylase is a high-Trp protein that well exemplifies an exported virulence factor that interacts with the host system under conditions of rapid growth. In *C. trachomatis* histone methylase (encoded by CT737) has been reported to methylate three different host proteins (Pennini et al., 2010). Not only its synthesis, but its export, is likely sensitive to Trp availability since its export depends upon the complex type III secretion system, some components of which exhibit high-Trp content. This methylase also undergoes self-methylation as a mechanism of increasing catalytic efficiency, a property of considerable interest in that the SAM substrate is expected to be of limited abundance under conditions of Trp starvation since the SAM transporter is a high-Trp protein.

Multiple paralogs of Pmp's are *Chlamydiaceae*-specific proteins, whose extruded N-termini are important virulence factors. Although these N-termini extensions, in fact, have very low Trp content, their extrusion depends upon a C-terminal component that is a transmembrane barrel autotransporter of high Trp content (Henderson and Lam, 2001). The C-terminal portion of Pmp's illustrate how proteins that have Trp “hotspots” can be sensitive to Trp depletion without having an overall high Trp content. In the case of the multiple Pmp paralogs, which are specific to the *Chlamydiaceae* and therefore are not subject to extra-Family homolog comparisons, Up-Trp selection seems intuitively obvious in consideration of the unusual density of C-terminal Trp hotspots.

### Key metabolic steps

PEP carboxykinase is very much a key protein and is highlighted in Figure 2, not only because it is a conspicuous Trp-rich protein but because it is a touchstone element operating at the center of a complex and interactive chain of vulnerability to Trp depletion. Its GTP and oxaloacetate substrates require Trp-rich transporters. The PEP product of the enzyme reaction is a crucial substrate for multiple pathways that include cell division, lipopolysaccharide biosynthesis, and menaquinone biosynthesis. PckG was suggested to contribute strongly to an “Achilles-heel vulnerability” in the cell-division pathway at the level of MurA, the initial specific step of the Lipid II pathway of cell division (Lo et al., 2012). In addition to PEP, MurA requires N-acetyl-glucosamine as a co-substrate. N-acetyl-glucosamine, in turn, is a reaction product of GlmV, a Trp-rich enzyme that also utilizes UTP for catalysis. Furthermore, MurA is in competition with two other enzymes for N-acetyl-glucosamine, one being the initial step of LPS biosynthesis, and the other a downstream enzyme of cell division (MurG). Thus, even though MurA has a Trp content which is only average, the availability of its substrates depends upon a multiplicity of other proteins of very high Trp content.

The so-called “cell wall anomaly” in chlamydiae (Moulder, 1993), states that although the organism has the genetic capacity to produce peptidoglycan monomers, it has no detectable peptidoglycan cell wall. Further, it may not make a canonical peptidoglycan structure, given the absence of any annotated transglycosylase enzymes to link the sugar moieties. These observations have given rise to speculations that a peptidoglycan-like structure is produced transiently at the septum during cell division. Brown and Rockey (2000) identified an antigen in apparent chlamydial division planes that was not proteinaceous and may possibly have been the peptidoglycan-like structure. Nutrient availability and other environmental conditions impact cell growth and division in bacteria, as reviewed by Hill et al. (2013). Recent publications (Gaballah et al., 2011; Ouellette et al., 2012) have suggested a critical role for MreB as a functional substitute for FtsZ (which is absent in *Chlamydiales*) in its role of organizing the division plane. We had previously noted (Lo et al., 2012) that it might be meaningful that *mreB* and *pckG* (encoding PEP carboxykinase) co-exist as overlapping genes in an apparent operon. This could potentially be extended to *snf*, an apparent operon component which is upstream of *mreB*. In view of the important relationship of PckG to cell division discussed above, this new information about the role of MreB in cell division is quite interesting. Snf is a putative helicase that, although neither Up-Trp nor Down-Trp, contains a Trp hotspot at its C-terminus (including a tandem WW motif). This could potentially destabilize the putative three-gene operon to effect an overall synergism of impact upon cell division.

## Reductive evolution of Trp biosynthesis in *Chlamydiales*

### Extreme phylogenetic variation of reductive evolution for *trp* genes

**Figure 3:**
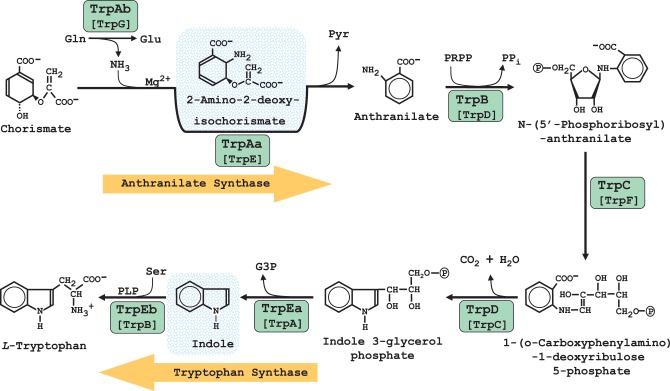
**Biochemical pathway of Trp biosynthesis.** The green ovals encircle the logical acronym designations which are based upon the order of enzyme reactions (Xie et al., 2003). Below the latter are shown the classic acronym designations. Enzyme-bound intermediates for anthranilate synthase and tryptophan synthase are shown with blue shading. The initial pathway reaction is catalyzed by anthranilate synthase. The TrpAa subunit can catalyze the reaction as an aminase utilizing ammonia as a nitrogen donor, but the physiological reaction is carried out as an amidotransferase via the contribution of the TrpAb subunit which utilizes glutamine as the source of the ammonia reactant. Although tryptophan synthase is a two-subunit complex in which indole is an enzyme-bound intermediate, isolated TrpEb is capable of utilizing free indole.

The genes of Trp biosynthesis have generally undergone reductive evolution throughout most of the *Chlamydiales* Order. See Figure 3 for the reactions of Trp biosynthesis, together with the acronyms that were originally formulated for *E. coli* shown in comparison with the logical set of replacement acronyms used here in which enzymes were named in the order of pathway steps (Xie et al., 2003). The *Chlamydiaceae* vary considerably in the extent to which genes encoding the enzymes of Trp biosynthesis have resisted reductive evolution. *C. abortus, C. pneumoniae*, and *C. psittaci*, have lost all *trp* genes. (Note that in accordance with the opinion of many chlamydiae experts (Stephens et al., 2009) that all known species so far belong to the single genus *Chlamydia*, we do not use the *Chlamydophila* genus designation in current use by NCBI). The genome of *C. muridarum* has retained only a *trpC* remnant that appears to have lost important catalytic residues (Xie et al., 2002). Some of the other chlamydiae have partial-pathway remnants that are no longer connected to chorismate, but which have evolved some fascinating functional specializations as detailed below. Table 1 lists all of the *Chlamydiales* genes of *trp* biosynthesis and provides hyperlinks to the SEED database (Overbeek et al., 2005). This affords convenient scrolling among adjacent genes and quick access to SEED tools.

**Table 1:** **Proteins of Tryptophan biosynthesis in *Chlamydiae***.

**Organism^a^**	**Protein acronyms, Trp content^d^, NCBI gene numbers, and SEED identifiers**								
	**TrpR**	**TrpL**	**TrpAa**	**TrpAb**	**TrpB**	**TrpD**	**TrpC**	**TrpEb**	**TrpEa**
***Simkania negevensis* Z**	**1/91**	NP	**0/490**	**0/193**	**1/330**	**0/264**	**1/207**	**2/389**	**1/253**
NCBI gene number	SNE_A10150		SNE_A10160	SNE_A10170	SNE_A10180	SNE_A10190	SNE_A10200	SNE_A10210	SNE_A10220
SEED protein page	fig|331113.3.peg.1081		fig|331113.3.peg.1082	fig|331113.3.peg.1083	fig|331113.3.peg.1084	fig|331113.3.peg.1085	fig|331113.3.peg.1086	fig|331113.3.peg.1087	fig|331113.3.peg.1088
***Chlamydia caviae* GPIC^b^**	**1/102**	NP	NP	NP	**2/305**	**0/274**	**2/207**	**2/392**	**0/258**
NCBI gene number	CCA00562				CCA00563	CCA00564	CCA00565	CCA00566	CCA00567
SEED protein page	fig|227941.6.peg.606				fig|227941.6.peg.607	fig|227941.6.peg.608	fig|227941.6.peg.609	fig|227941.6.peg.610	fig|227941.6.peg.611
***Chlamydia felis* Fe/C-56**	**1/102**	**2/39**	NP	NP	**2/318**	**0/275**	**2/207**	**2/391**	**0/258**
NCBI gene number	CF0440	CF0441			CF0439	CF0438	CF0437	CF0436	CF0435
SEED protein page	fig|264202.11.peg.459	fig|264202.11.peg.458			fig|264202.11.peg.457	fig|264202.11.peg.456	fig|264202.11.peg.455	fig|264202.11.peg.454	fig|264202.11.peg.453
***Chlamydia pecorum* E58**	**1/93**	NP	NP	NP	**2/327**	**0/290**	**2/208**	**2/393**	**0/256**
NCBI gene number	G5S_1088				G5S_1087	G5S_1086	G5S_1085	G5S_1084	G5S_1083
SEED protein page	fig|331635.3.peg.973				fig|331635.3.peg.972	fig|331635.3.peg.971	fig|331635.3.peg.970	fig|331635.3.peg.969	fig|331635.3.peg.968
***Chlamydia trachomatis* D/UW-3/CX**	**1/95**	**3/50**	NP	NP	NP	NP	**3/208**	**2/392**	**0/253**
NCBI gene number	CT169	CT169a					CT327	CT170	CT171
SEED protein page	fig|272561.5.peg.182	fig|272561.5.peg.969					fig|272561.5.peg.352	fig|272561.5.peg.183	fig|272561.5.peg.184
***Chlamydia muridarum* Nigg**	NP	NP	NP	NP	NP	NP	**3/209**	NP	NP
NCBI gene number							TC065		
SEED protein page							fig|243161.6.peg.642		
***Coxiella burnetii* Dugway 5J108-111^c^**	**1/91**	NP	**0/493**	**0/121**	**0/334**	**0/258**	**1/601**	**1/601**	**0/268**
NCBI gene number	CBUD_1566		CBUD_1249	CBUD_1249a	CBUD_1251	CBUD_1252	CBUD_1253	CBUD_1253	CBUD_1255
SEED protein page	fig|434922.5.peg.1563		fig|434922.5.peg.1259	fig|434922.5.peg.1260	fig|434922.5.peg.1261	fig|434922.5.peg.1262	fig|434922.5.peg.1263	fig|434922.5.peg.1263	fig|434922.5.peg.1264

a The following have no Trp pathway genes: Chlamydia abortus, C. pneumoniae, C. psittaci, Protochlamydia amoebophila, Parachlamydia acanthamoebae and Waddlia chondrophila.

b Chlamydia caviae has a gene (CCA00559) encoding a duplicate TrpEb (0/413) fig|227941.6.peg.600.

c Coxiella burnetii has the fusion TrpC·TrpEb.

d Number of Trp residues per amino-acid length shown as numbers with bold fonts.

### Does *Simkania* retain the ancestral *trp* operon?

Only *Simkania negevensis* (Sneg) possesses a complete Trp pathway, in fact being in possession of the complete multi-branched pathway that extends to all three aromatic amino acids. Sneg deploys a very compact *trp* operon *trpR/trpAa/trpAb/trpB/trpD/trpC/trpEb/trpEa/aroA*. Only 18 nucleotides separate *trpR* and *trpAa*, and all other adjacent genes overlap indicating translational coupling. The inclusion of the *trpR* repressor gene within the operon indicates the existence of a form of self-regulation called autoregulation (Merino et al., 2008). The C-terminal *aroA* member of the operon encodes one of the three 2-keto,3-deoxy-**D**-*arabino*-heptulosonate-7-P (DAHP) synthase paralogs present in the genome, all belonging to the AroA_*I*β_ superfamily (Jensen et al., 2002). The operonic DAHP synthase is probably specialized to ensure precursor provision to the Trp pathway, similar to the classic situation of partitioned AroA isoenzymes in *E*. *coli* (Ahmad et al., 1986). Although Sneg appears to sustain a complete, intact pathway of Trp biosynthesis, it shares a number of Up-Trp and Down-Trp selections that are common to the *Chlamydiales*, as elaborated later. This suggests that acquisition of Trp from host resources was important at a stage that preceded reductive evolution of *trp* genes.

### Variant linkages of chorismate to menaquinone and aromatic amino acids

Sneg is thus far unique among the chlamydiae in utilization of chorismate as a precursor that feeds into biosynthesis of Trp, tyrosine, and phenylalanine—as well as into menaquinone biosynthesis via the classic isochorismate (DH2N) pathway. *Waddlia* and Paca/Pamo have retained the chorismate-to-DH2N menaquinone pathway, but this now appears to be a linear pathway composite instead of a branched pathway since the Trp, tyrosine and phenylalanine branches have all been lost. Although the *Chlamydiaceae* also possess a linear chorismate-to-menaquinone pathway, the menaquinone pathway is the newly discovered DH6N variation as discussed fully in a later section.

### The Trp/kynurenine/Trp cycle

*C*. *caviae*, *C*. *felis*, and *C*. *pecorum* have a nearly complete Trp pathway that lacks the initial anthranilate synthase step. These species are able to implement an alternative synthesis of anthranilate from kynurenine, a host metabolite produced from host Trp in two steps following the IDO reaction. This was originally described by Xie et al. (2002) for *C*. *caviae* (at that time called *C*. *psittaci*). This group of organisms has a novel *trp* operon (*trpR/trpB/trpD/trpC/trpEb/trpEa/kynU/kprS*). This encodes all enzymes of Trp biosynthesis, except for the two subunits of anthranilate synthase (TrpAa and TrpAb). This discontinuity effectively disconnects the Trp pathway from the chorismate biosynthesis pathway and requires a different source of anthranilate. The intergenic spacing between *trpR* and *trpB* is much greater in these organisms (about 230 nucleotides) than that observed between *trpR* and *trpAa* in Sneg, indicating that the loss of trpAa/trpAb occurred in a way that moved *trpR* further away from the succeeding gene of the operon. This potentially provides space for regulatory features. Indeed, in C. *felis* a gene encoding a potential *trpL* leader peptide that has tandem Trp residues (MKINKADTFSTNALALLNNLCALYSSAFPFFFSL**WW**AFAQ) is located between *trpR* and *trpB* (see Table 1). Attenuator structures have not been reported for this region in the *caviae/felis/pecorum* grouping, and thus whether repression control by *trpR* may be integrated with attenuation control is a possibility that awaits experimental work. The last two genes of the operon are thus far not found elsewhere in the chlamydiae, nor are they known to comprise part of any other *trp* operon. *kynU* encodes kynureninase, catalyzing the formation of anthranilate from host kynurenine (a catabolite of Trp). This reaction allows host-diverted Trp to be recycled (via the interception of host kynurenine) back to Trp in the pathogen. PRPP synthase is the gene product of *prsA*. It is needed to produce PRPP, a co-substrate with anthranilate in the reaction catalyzed by TrpB. *prsA* is the other unique gene member of the operon and is closely related to *prsA* present in other chlamydiae genomes. In contrast, *kynU* is not present thus far in any other chlamydiae and may have originated via lateral gene transfer. Although a mammalian donor seemed feasible, detailed bioinformatic work did not confirm this possibility (Xie et al., 2002).

The traditional enzymes of Trp biosynthesis shown in Table 1 all have a low Trp content, as indeed is generally expected because of selection for low cognate amino acid bias in amino-acid biosynthetic enzymes (Alves and Savageau, 2005). However, it has been suggested (Lo et al., 2012) that the recycling mechanism is unlikely to function for Trp production under conditions of persistence. Rather than being a seemingly obvious mechanism to thwart the host strategy of Trp depletion, the recycling mechanism may be geared to jump-start vegetative pathogen growth during the transitional phase where persistent cells encounter renewed availability of host Trp. This interpretation was based upon the fact that kynureninase is a protein of very high Trp content, as well as upon the consideration that PRPP synthase utilizes ATP (scarce under conditions of persistence) in a reaction in which two high-energy equivalents are consumed.

### Indole utilization

Yet another Trp partial-pathway consisting only of gene products encoded by the operon assemblage *trpR/trpEb/trpEa* is maintained by *C. trachomatis* (Ctra). *C. trachomatis* also has a *trpC* pseudogene remnant, which likely is not functional (Xie et al., 2002). A single enzyme activity, the condensation of indole and serine to produce Trp, is a catalytic property of TrpEb. TrpEb is one of two subunits of Trp synthase, a protein/protein complex which catalyzes an overall reaction in which indole is an enzyme-bound intermediate produced by the TrpEa half-reaction and utilized by the TrpEb half-reaction (Figure 3). It is interesting that the source of indole is not the human host, but rather comes from indole-producing organisms that can co-exist in the tissue niche occupied by genital strains. Thus, indole can rescue genital strains of Ctra—but not ocular strains—from the Trp starvation caused by the host-mediated induction of IDO (Fehlner-Gardiner et al., 2002). Hence, strain-specific host tropism corresponds with niche-specific ability to scavenge indole from a given co-existing microbial community. It is curious that TrpEb function in Ctra requires the concomitant presence of a full-length, catalytically inactive TrpEa subunit (Fehlner-Gardiner et al., 2002). It seems likely that TrpEa may be required for stabilization of TrpEb since these subunits have well-established protein-protein interactions (Xie et al., 2002). This atypical interactive requirement for TrpEb function in Ctra is not a general characteristic of chlamydial TrpEb proteins, judging from the observed ability of isolated TrpEb from *C. caviae* to carry out the indole-plus-serine condensation in the absence of TrpEa (Wood et al., 2004).

Regulation by *trpR* in Ctra has been demonstrated by Akers and Tan (2006) in a publication that cites the report by Merino and Yanofsky (2005) of an attenuator just upstream of *trpEb*. The attenuation mechanism was included as an important aspect of the Akers and Tan model. In the same year Carlson et al. (2006) inexplicably asserted that no attenuator could be found upstream of *trpEb*. Subsequently, the existence of a predicted transcription attenuator between *trpR* and *trpEb* was affirmed (Merino et al., 2008). Indeed this attenuator was also found to be followed by a putative *trpL* gene encoding a leader peptide. The leader peptide has 59 residues in which the C-terminal segment has a Trp-rich hotspot of 3/12 Trp residues (MHALLMNKYSVLAVLVHKYSCSMPCKSAFQADCFQDIQKFILLQRA**W**LSFES**W**RLST**W**R). The predicted secondary structure can be accessed at Merino's website http://cmgm.stanford.edu/%7Emerino/Chlamydia_trachomatis/15604889.html. Thus, there appears to be a strong basis for the TrpR repression mechanism to be supplemented by a transcription attenuation mechanism, as is the case in *E. coli*. In the latter case, attenuation increases the range of regulation mediated by TrpR by an additional order of magnitude (Merino et al., 2008).

### *Coxiella Burnetii*: lateral gene transfer (LGT) recipient of the *Simkania trp* operon?

*Coxiella* organisms are *Gamma*-*proteobacteria* that are obligate intracellular pathogens of humans and other animals. It appears to have lost competence for Trp biosynthesis, judging from the pseudogene character of both *trpAb* and *trpB* (Xie et al., 2003). This appears to be a recent ongoing process of reductive evolution since most of the genes remain largely intact, and all of them are still recognizable. Interestingly, the structural genes and *trpR* are close homologs of those from *Simkania*, rather than of those from other *Gamma*-*proteobacteria*. The operon construction (see bottom of Table 1) differs in that (i) *trpR* (the initial operon gene of *Simkania*) has become separated from the *trp* operon in *Coxiella*, (ii) *trpC* and *trpEb* are fused in *Coxiella*, and (iii) the *Coxiella* operon does not contain *aroA* (the last operonic gene of *Simkania*). If this *aroA* gene was acquired by *Coxiella* via LGT, it was not retained since all *Coxiella aroA* paralogs are closely related to those of other *Gamma-proteobacteria*. Because the homology relationship of the *trp* genes of *Coxiella* are not phylogenetically congruent with those of other *Gamma*-*proteobacteria*, whole-pathway LGT from a *Chlamydiales* donor to a *Coxiella* recipient, is implicated—a phenomenon described for *trp* operons in a number of other cases (Xie et al., 2003).

## Which events of Up-Trp and Down-Trp selection preceded divergence of *Chlamydiaceae*?

**Figure 4:**
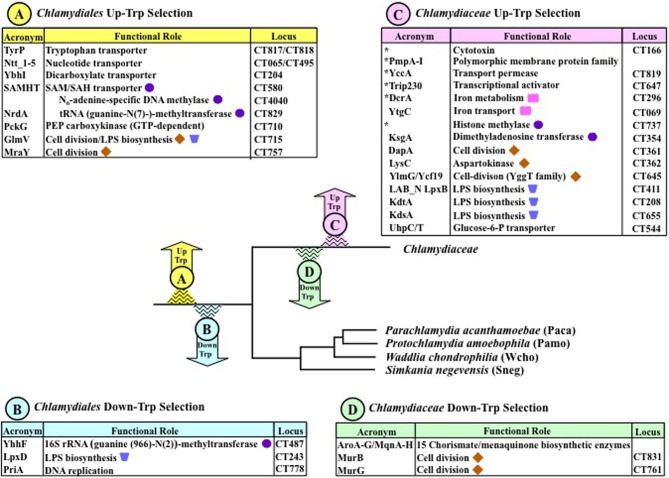
**Events of Up-Trp and Down-Trp selection in phylogenetic context.** Early evolutionary events, i.e., ancestral to the *Chlamydiales* Order, are show in yellow (Up-Trp) and blue (Down-Trp). Later events specifically ancestral to the *Chlamydiaceae* are shown in pink and green. Even more recent events distinguish *C. trachomatis* from *C*. *muridarum* as previously exemplified in Figure 1. Different steps of several functional processes (cell division, LPS biosynthesis, and methylation) are marked with identifying symbols Asterisks within the box at the upper right indicate gene products that are expressed in the *Chlamydiaceae* but not elsewhere in the *Chlamydiales* Order.

The list of proteins identified as Up-Trp or Down-Trp proteins can be sorted into groups that have undergone Trp-content selection at different evolutionary times. Those occurring at either the taxon level of the *Chlamydiaceae* or at the deeper taxon level of the *Chlamydiales* are enumerated in Figure 4.

### *Chlamydiales* Up-Trp selection

The *Chlamydiales* ancestor (yellow arrow) underwent Up-Trp selection for a number of important transporters. These include permeases for nucleotides, dicarboxylate substrates, S-adenosylmethionine (SAM), and for transport of Trp itself via TyrP, as listed in section A at the upper left of Figure 4. CT numbers for the *C. trachomatis* genes are given at the right for convenient reference. Paralog expansion of TyrP, which occurred extensively throughout the *Chlamydiales* is discussed in detail in a later section. In the *Chlamydiaceae*, two paralogs accommodate nucleotide transport, as documented fully for the *Chlamydia* genus (Tjaden et al., 1999). One catalyzes ATP/ADP exchange, and the other facilitates the import of RNA nucleotides. A recent update of nucleotide parasitism at the deeper phylogenetic level of the *Chlamydiales* order highlights the individuality of the nucleotide transporters in the chlamydiae with respect to paralog number, transport specificity, and molecular mechanism—albeit against a general background of overall similarity (Knab et al., 2011). Families other than the *Chlamydiaceae* exhibit the most extensive paralog expansion. In one well-studied example, *P*. *amoebophila* (Pamo) possesses five nucleotide transporters. PamNTT1 is an ATP/ADP antiporter; PamNTT2 is a nucleoside triphosphate antiporter balancing the nucleotide pool; PamNTT3 is a UTP/H^+^ symporter; PamNTT4 transports NAD^+^ in exchange for ADP; and PamNTT5 is a GTP/ATP/H^+^ symporter (see (Knab et al., 2011) and refs. therein). Sneg has four nucleotide transporters, the substrate specificity of one being uncertain. The two *C. trachomatis* (Ctra) nucleotide transporters utilize a cumulative 31 Trp residues for minimal monomer units. This is a very substantial Trp content, but the Trp burden associated with nucleotide transporters is even greater in the remaining Families of the *Chlamydiales*. Thus, the Trp burden is a cumulative 52 Trp residues for the five Pamo proteins, 62 cumulative Trp residues for the five *Waddlia chondrophila* (Wcho) proteins, and 47 cumulative Trp residues for the four Sneg proteins.

Up-Trp selection for the SAM-dependent DNA methylase and the SAM-dependent tRNA methyltransferase suggest a degree of synergistic sensitivity to Trp availability since not only these proteins *per se*, but the availability of their SAM substrates is reduced in the absence of Trp. This is because the SAM/SAH transporter (Binet et al., 2011) is conspicuously high in Trp content. This dependence of the methylase and the methyltransferase upon the SAM transporter is indicated in Figure 4 by indenting their names under that of the SAM/SAH transporter.

The intricate role of PEP carboxykinase in the overall metabolic network has been discussed above. The evolutionary choice of *pckG*, which uses GTP rather than of *pckA* which uses ATP, exemplifies the exercise of preference for one of two non-homologous functional equivalents that characteristically has a distinctly high Trp content. The function of PEP carboxykinase as the source of PEP is linked to several complex pathways that require the input of PEP. The high Trp content of several enzymes that participate in cell division (GlmV and MraY) or in LPS biosynthesis (GlmV) is one factor which should result in at least some restraint of these processes under conditions of Trp limitation. The additional impact of substrate limitation must be substantial. For example, the cell-division pathway requires 1 PEP, 1 UTP, and 5 ATP substrate inputs (see Figure 7 of Lo et al., 2012).

### *Chlamydiales* Down-Trp selection

The 16S rRNA methyltransferase (YhhF) and LpxD (shown at the lower left in Figure 4) are cases of Down-Trp selection that probably exemplify a compensatory Down-Trp selection in pathway functions of the *Chlamydiales* Order where one or more Up-Trp selections discussed above and listed at the upper left of Figure 4 have probably created translation hurdles. Such a mechanism can help offset the great metabolic expense of using Trp. PriA is a very large molecule engaged in DNA replication. The extensive Down-Trp selection of PriA throughout the *Chlamydiales* supports its significance generally for survival under conditions of Trp limitation in the *Chlamydiales* and for maintenance of the persistent state in *Chlamydiaceae* in particular.

### *Chlamydiaceae* Up-Trp selection

The *Chlamydiaceae* have experienced a great intensification of Up-Trp selection that extends beyond those covered above for the *Chlamydiales*, as summarized in Box C at the upper right of Figure 3. Generally, these selections can be rationalized in a context of co-evolution with properties of the mammalian host. In addition to the two Up-Trp selections in the *Chlamydiales* that are relevant to cell division, three additional Up-Trp selections (DapA, LysC, and YlmG/Ycf19) are *Chlamydiaceae*-specific. Similarly, in addition to the two Up-Trp selections in the *Chlamydiales* that are relevant to LPS biosynthesis, three additional reinforcing Up-Trp selections (KdtA, KdsA, and LAB_N LpxB) are *Chlamydiaceae*-specific. (The appending of the Trp-rich LAB_N domain by fusion to LpxB exemplifies one mechanism for conferring increased vulnerability to Trp starvation.) The glucose-6-P transporter (UhpC/T) is a fusion protein that contains both receptor (UhpC) and transport (UhpT) functions, which are separate in other organisms. This protein is always Trp-rich in nature, but it was deemed to have been subject to Up-Trp selection in *Chlamydiaceae* because of further elevated Trp content with the addition of multiple Trp hotspots (Lo et al., 2012).

A number of important high-Trp proteins in the *Chlamydiaceae* are generally absent elsewhere in the *Chlamydiales* (shown with asterisks in Figure 4). These include the paralog family of Pmp's A-I, DcrA involved in iron metabolism, a histone methylase, an uncharacterized transport permease (YccA), a putative transcriptional activator (Trip230), and cytotoxin. The Pmp's, the histone methylase, and the cytotoxin were discussed earlier. The Trip230 activator is likely to be highly sensitive to Trp limitation since its Trp content scored the highest p/P Trp ratio in the *Chlamydiaceae* proteomes. As a probable transcriptional activator suggested to be involved in folate metabolism (Lo et al., 2012), it could be engaged in master/slave relationships in which effects of Trp limitation upon Trip230 may affect multiple other proteins, regardless of their individual Trp contents.

### *Chlamydiaceae* Down-Trp selection

The increased *Chlamydiaceae*-specific expenditure of Trp for the cell-division pathway shown in box C at the upper right of Figure 4 is offset by the *Chlamydiaceae*-specific Down-Trp selection of MurB and MurG (box D). A most striking Down-Trp selection has occurred for the 15-step pathway that converts erythrose-4-P and PEP to menaquinone via chorismate. This suggests that function of this pathway is particularly crucial for maintenance of the persistent state. Menaquinone biosynthesis is covered comprehensively in a later section.

## The highly expansive distribution of TyrP orthologs and paralogs in *Chlamydiales*

### Chlamydiaceae

**Figure 5:**
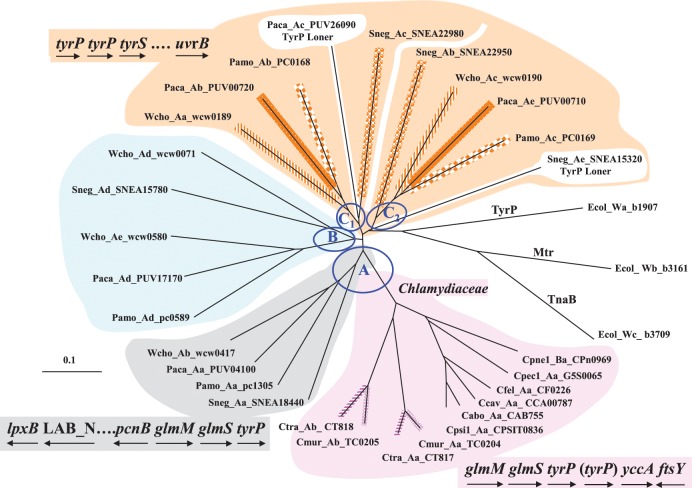
**Multiplicity of the TyrP transporter for Trp in the *Chlamydiales* Order.** The three *E*. *coli* paralogs for Trp transport are shown at the right. Three main ortholog clusters of TyrP proteins (designated as A–C) are evident, together with an admixture of a few additional paralogs. Cluster A contains one TyrP protein from Wcho, Paca, Pamo, and Sneg – as well as the divergent set of proteins from the *Chlamydiaceae*. Ctra and Cmur each have two paralogs, which appear to have occurred independently via gene duplications that followed the speciation divergence. At the lower left is shown a suggested ancestral gene neighborhood which can be compared to the *Chlamydiaceae* gene neighborhood shown at the lower right. In the latter gene neighborhood inclusion of “(*tyrP*)” is relevant only to the gene duplicate present in the *Cmur/Ctra* pair. The *Chlamydiaceae* have no representation in ortholog clusters B and C. Cluster B possesses members from Wcho, Sneg, Paca, and Pamo – with Wcho having an additional paralog member. Cluster C is comprised of tandem *tyrP* genes (see the gene neighborhood at the upper left) which encode the four sets of ortholog pairs shown (with matched line patterns). Thus, duplication of the ancestral Group-C ortholog in the common ancestor of Wcho, Sneg, and Paca/Pamo generated the paralog sets that are positioned within Group C_1_ and C_2_. In addition Paca and Sneg possess a third paralog belonging to cluster C; the genes encoding these are unlinked to the aforementioned tandem genes (hence their gene products being designated as “TyrP Loners”). Abbreviations for organisms populating the *Chlamydiaceae*: Ctra, *Chlamydia trachomatis*; Cmur, C. *muridarum*; Cpsi, *C. psittaci*; Cabo, *C*. *abortus*; Ccav, *C*. *caviae*; Cfel, *C*. *felis*; Cpec, *C*. *pecorum*; and Cpne, *C*. *pneumoniae*. Other *Chlamydiales* organisms are abbreviated as given in Figure 4.

The TyrP family of permeases transport Trp and/or tyrosine with varying specificity (Sarsero et al., 1991). This is exemplified in *E. coli* where three permeases are utilized: TyrP being specific for tyrosine (Ecol_Wa_b1907 in Figure 4), Mtr having high affinity for Trp transport (Ecol_Wa_b3161), and TnaB having low affinity (but high capacity) for Trp transport (Ecol_Wc_b3709). Members of the *Chlamydiaceae* generally possess a single *tyrP* gene represented in Group A of Figure 5, but recent gene duplications have occasionally generated paralogs. Both *C. trachomatis* and *C. muridarum* possess paralog *tyrP* genes in a tandem configuration. It is interesting that *tyrP* copy number may be relevant to tissue tropism in *C*. *pneumoniae* at the strain level since respiratory strains, but not vascular strains, were found to have two or more paralogous copies of *tyrP* (Gieffers et al., 2003). It is very clear that induction of persistence occurs in *C. pneumoniae* as a result of IFN-γ-mediated activation of host cells (reviewed in Roulis et al., 2013), and although the regulation of Up- and Down-Trp protein expression has not been evaluated for *C. pneumoniae*, novel transcriptional patterns have been reported to be based on the method of persistence induction (Roulis et al., 2013). It was, however, noted in earlier work (Dairi, 2009) that a lesser capacity for Trp import, assumed to be associated with single *tyrP* copies in *C. pneumoniae*, may be tied to a greater tendency to exist in the persistent state. *tyrP* copy number might also be relevant to tissue tropism of *C. trachomatis* since an oculotropic trachoma isolate was found to have one disrupted copy of the tandem *tyrP* genes present in genitotropic strains (Carlson et al., 2005).

### Distribution of TyrP genes in *Chlamydiales*

In contrast to the *Chlamydiaceae*, the *tyrP* family of genes exhibits extensive expansion in other Family taxa of the *Chlamydiales*, as illustrated by the radial tree shown in Figure 5 where three ortholog groups are designated as Groups A, B, and C. Groups A and C are associated with the conserved gene neighborhoods shown. Only Group-A TyrP proteins are found in the *Chlamydiaceae* and this is probably encoded by the ancestral gene, this ortholog being the only one that is present in each member of the four chlamydial families. The subdivision of Group A into two groups (*Chlamydiaceae* in pink on the right) and members of the other three families (Wcho, Paca, Pamo, and Sneg in gray on the left) corresponds with the variation of gene neighborhood shown. The gene order *glmM glmS tyrP* is absolutely conserved in all of the *Chlamydiales*. In the *Chlamydiaceae* gene neighborhood *yccA* encodes an uncharacterized protein that may participate in cell division since its homolog in *E. coli* has been reported to interact with FtsH, and it has a very high Trp content. *ftsY* encodes a signal recognition particle that is relevant to cell division. *glmM* and *glmS* encode initial enzymes leading to synthesis of UDP-N-acetylglucosamine, a crucial metabolite that is located at a branchpoint that diverges to LPS biosynthesis, on the one hand, and to the Lipid II pathway for cell division, on the other hand. The gene order on the lower left is probably very similar to the ancestral arrangement, perhaps including *yccA* and *ftsY* following *tyrP* since these genes can still be found in the vicinity of Group-A *tyrP* in Wcho, Paca, Pamo, and Sneg. Following the divergence of *Chlamydiaceae* from the other Families, *Lab_N* underwent fusion with the adjacent *lpxB* to give fused *Lab_N/lpxB* gene which is present in all *Chlamydiaceae*. This fusion event was associated with a translocation event that separated *Lab_N/lpxB* a substantial distance from the ancestral gene neighborhood.

The *Simkaniaceae*, *Parachlamydiaceae*, and *Waddliaceae* Families are all represented by at least one member in Groups B and C TyrP's. Wcho has two Group-B TyrP paralogs. The greatest paralog expansion has occurred in Group C. A gene duplication in the common ancestor of the three families has generated tandem paralog sets that are adjacent to *tyrS* and in the vicinity of *uvrB*. In Figure 4 Wcho, Paca/Pamo, and Sneg each are represented by the set of two paralogs, one in Group C_1_ and the other in Group C_2_. In addition Paca has an additional paralog that emerged from Group C_1_, and Sneg has an additional paralog that emerged from Group C_2_. The latter two are referred to as TyrP loners because the encoding genes have been translocated far from the parental paralog pairs. The Sneg gene neighborhood has been disrupted somewhat from the suggested ancestral arrangement in that the two paralog *tyrP* genes have been separated by two inserted hypothetical genes, and they are far separated from *tyrS*, as well. *uvrB* is still a close-neighbor gene.

### Lysosomal degradation as a source of Trp

It has recently come to be appreciated (Ouellette et al., 2011) that in order for Trp import to be fully understood, an evaluation of oligopeptide or Trp molecules obtained from ongoing degradation processes in host lysosomes must be included. Not only is the pathogen inclusion physically proximal to lysosomes, but a substantial multiplicity of oligopeptide and dipeptide transporters occurs in chlamydial genomes. For example, Ctra possesses, as just one of many illustrative cases, an *oppABC* operon (CT478-CT480) encoding three gene products having a total Trp burden of 30 Trp residues (for monomeric entities). Peptidase gene products are represented as well. This aspect of Trp acquisition awaits detailed bioinformatic analysis.

## *Chlamydiaceae* are the sole taxon family within *Chlamydiales* to deploy the recently recognized DH6N pathway of menaquinone biosynthesis

**Figure 6:**
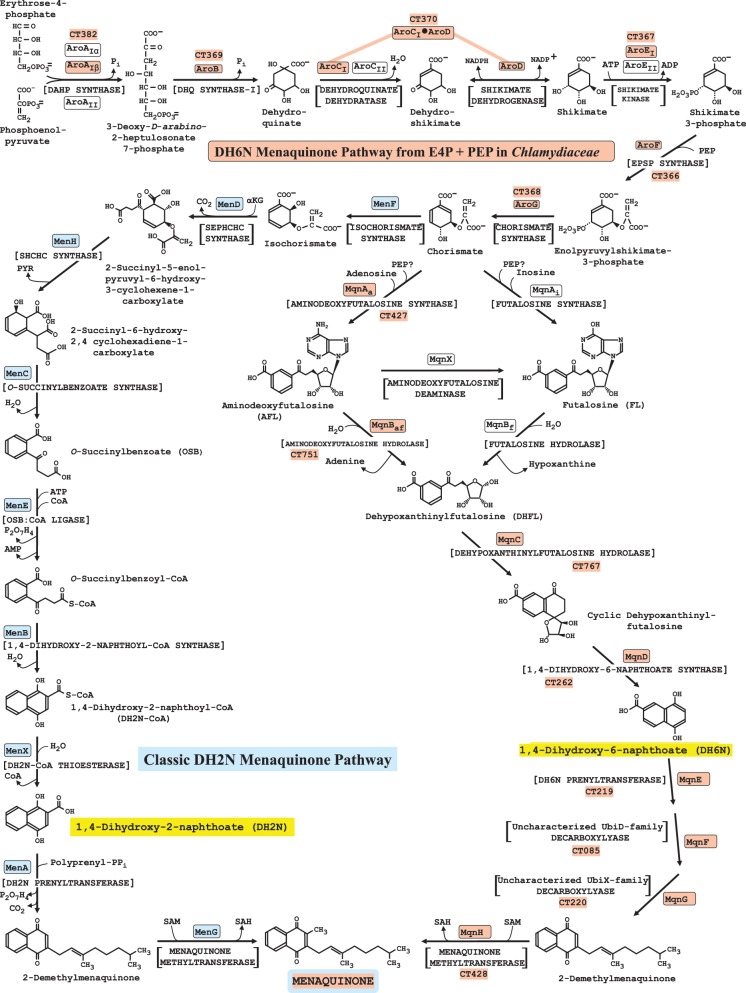
**Variant menaquinone pathways in nature.** The composite diagram shows the biochemical variations to menaquinone that are so far known to exist in nature. The proposed 15-step *Chlamydiaceae* pathway, which includes the 7-step pathway to chorismate, is indicated with orange boxes surrounding the enzyme acronyms. For reference, the encoding CT gene numbers for *C. trachomatis* D/UW-3/CX are also shown. Multiple acronyms are indicated for the three enzyme steps in the chorismate pathway that can be performed by distinct sub-homolog types or by non-homologous isofunctional analogs (see http://www.aropath.lanl.gov/ for the logical system of acronym assignment used). AroC_*I*_ and AroD are domain components of a single protein encoded by fused genes. The classic dihydroxy-2-naphthoate (DH2N) pathway from chorismate, generally known as the isochorismate pathway of menaquinone biosynthesis, is shown with acronyms in blue boxes at the left. The alternative DH6N pathway shown at the right was originally called the futalosine pathway (Dairi, 2009, 2012), but futalosine has proven to not necessarily be used as an intermediate because of alternative early-pathway steps that exist. Therefore, we refer to this as the DH6N pathway. The nine enzymes of the DH6N pathway in *S. coelicolor* are encoded by SCO4506, SCO4662, SCO4327, SCO4550, SCO4326, SCO4491, SCO4490, SCO4492, and SCO4556. The DH2N and DH6N structures, for which the pathways are named, are highlighted in yellow.

*In E. coli* ubiquinone and menaquinone are essential components of the electron-transport chain under aerobic or anaerobic conditions, respectively. In many organisms only menaquinones are used (Bentley and Meganathan, 1982), and this appears to be the case in the *Chlamydiales.* The classic menaquinone pathway is illustrated with blue highlighting as part of the composite given in Figure 6. The thioesterase reaction of the classic pathway, previously thought to be perhaps a spontaneous, non-enzymatic transformation, has recently been documented as an enzymatic reaction (Widhalm et al., 2009) and is denoted as MenX in Figure 6.

### Comparison of the classic DH2N pathway with the DH6N pathway

Recently, an alternative pathway of menaquinone biosynthesis called the futalosine pathway by Dairi and his collaborators was reported (Hiratsuka et al., 2008; Dairi, 2009, 2012). Since futalosine has subsequently proven to not necessarily be an intermediate, this pathway is herein referred to as the DH6N pathway and the classic isochorismate pathway is referred to as the DH2N pathway. The *Chlamydiaceae* family is distinctive among the *Chlamydiales* in having the DH6N pathway. All other known *Chlamydiales* families use the classic DH2N pathway. As illustrated with yellow highlighting in Figure 6, DH6N and DH2N are closely related positional isomers. The newly recognized DH6N pathway itself exhibits variation in some of the early steps, such that futalosine is made directly following reaction of chorismate and inosine (denoted MqnA_*i*_in Figure 6) or indirectly in two steps by an initial reaction of chorismate and adenosine to form AFL (MqnA_*a*_) followed by a deaminase reaction (MqnX). A third flow route takes AFL directly to DHFL (MqnB_*af*_), thus by-passing futalosine altogether (Arakawa et al., 2011). Thus, a general MqnA reaction sorts into enzymes having specificity for adenosine (MqnA_*a*_) or having specificity for inosine (MqnA_*i*_). [Note that early tracer studies have indicated a likely role of PEP or pyruvate as a substrate reactant (Seto et al., 2008)]. Likewise, MqnB enzymes sort into those having specificity for aminodeoxyfutalosine (MqnB_*af*_) or for futalosine (MqnB_*f*_). Organisms such as *Thermus thermophilus* take the two-step futalosine pathway to DHFL, whereas organisms such as *Campylobacter*, *Helicobacter*, and *Chlamydia* use the two-step aminodeoxyfutalosine pathway to DHFL. Yet other organisms, such as *Streptomyces coelicolor* and *Acidothermus cellulolyticus* take the three-step pathway to DHFL, deploying MqnA_*a*_, the MqnX deaminase, and MqnB_*f*_(Dairi, 2012). MqnB_*af*_ has been identified recently as synonymous with 5'-methylthioadenosine nucleosidase (MTAN) in *Campylobacter jejuni* (Li et al., 2011).

### Features of DH6N pathway variation in *Chlamydiaceae*

The use of the adenosine-dependent step (MqnA_*a*_), rather than of the inosine-dependent step (MqnA_*i*_) by the *Chlamydiaceae* is consistent with the report (McClarty and Fan, 1993) that *C*. *psittaci* was able to utilize adenosine, but not inosine, from the host. Undoubtedly there are some interesting properties of the DH6N pathway that await discovery in the *Chlamydiaceae*. For example, CT263 is a gene of unknown function which is *Chlamydiaceae*-specific and which overlaps with CT262 encoding MqnD. CT261 encoding the epsilon subunit of DNA polymerase also overlaps CT262. In another operonic arrangement, CT427 encoding MqnA_*a*_ and CT428 encoding MqnH are contiguous, these genes encoding the first and last steps of the pathway. Curiously, CT426, an apparent paralog of CT767 encoding MqnC, is also a member of the operon. The enzyme encoded by CT426 is not shown as a catalytic participant in Figure 6, but the co-existence of two *mqnC* paralogs in nature is highly conserved. For example, *S. coelicolor* possesses in addition to the SCO 4550 *mqnC*, a sister *mqnC* paralog SCO4494 (32% identity of the gene products). These paralogs are members of the Radical_SAM family. The role of the paralog might have some sort of functional relationship with the assertion that PEP or pyruvate must be utilized in the early part of the pathway (Seto et al., 2008). The CT219 and CT220 genes overlap by 3 bp, and probably comprise an additional operon, another relationship which appears to be conserved (e.g., the corresponding genes in *S. coelicolor* are SCO4491 and SCO4492). CT 219 and CT220 have been annotated as *ubiA* and *ubiX*, respectively. However, the prenyltransferase, methyltransferase, and decarboxylase enzymes that exist in the ubiquinone and menaquinone pathways are homologs and can easily be mis-annotated.

### Menaquinone genes located on the lagging strand of replication

**Figure 7:**
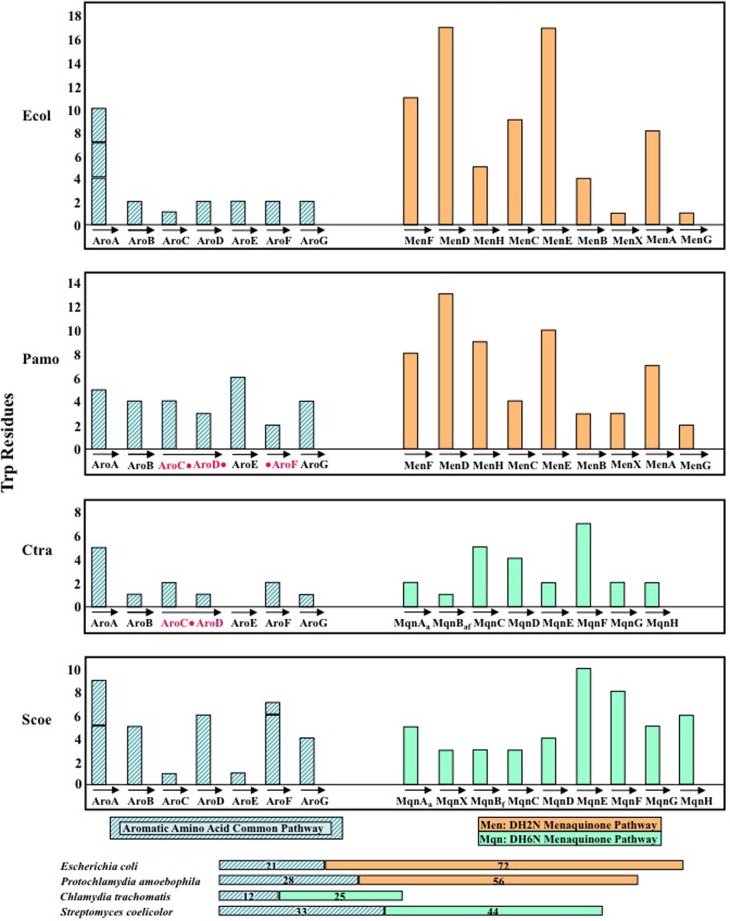
**Comparison of the Trp content of the chorismate/menaquinone pathway in *Chlamydia trachomatis* (Ctra), its close relative *Protochlamydia amoebophila* (Pamo), the classic bacterium *Escherichia coli* (Ecol), and *Streptomyces coelicolor* (Scoe).** Pamo and Ecol both utilize the classic DH2N pathway for menaquinone biosynthesis, and this is represented by the orange histogram bars. On the other hand, Ctra and Scoe both utilize the DH6N pathway, and this is indicated by the green histogram bars. Note by relating the acronyms under the histogram bars to the pathway diagrams in Figure 6 that Ctra and Scoe utilize different minor variations of the DH6N pathway, utilizing aminodeoxyfutalosine or futalosine, respectively, as unique intermediates. Ctra exhibits a fusion of AroC and AroD, whereas Pamo has a fusion of AroC, AroD, and AroF (as indicated in red). The cumulative total of Trp residues in the chorismate pathway and the connected menaquinone pathway are given within the color-coded horizontal bars displayed at the bottom of the figure.

A bias is well-known to favor the location of *trp* codons on the leading strand of replication. The cumulative influence of multiple *trp* codons in genes that encode high-Trp proteins should greatly increase the probability for the location of such genes to be on the leading strand. Location on the leading strand prevents head-to-head collisions of DNA polymerase engaged in DNA replication and RNA polymerase engaged in transcription. Of the 15 genes specifying the menaquinone pathway, 14 are located on the lagging strand of replication. This is consistent with the low *trp*-codon counts in these genes. The only exception is the initial *aroA* gene encoding DAHP synthase, a protein that does have a higher Trp content than other enzymes of the common aromatic pathway leading to chorismate (see Figure 7). Surprisingly, some crucial Ctra proteins of very high Trp content were observed to be encoded by genes located on the lagging strand of replication (Lo et al., 2012). These include the genes encoding glucose-6-P translocase, dicarboxylate transporter, and ADP/ATP translocase. It was suggested that perhaps transcripts of such genes might be stockpiled under conditions of persistence where their translation would not be favorable. During transition to rapid vegetative growth occasioned by the renewed presence of Trp, the availability of key transcripts might help jumpstart this developmental process. This idea is based upon the finding that transcription and translation are uncoupled in *Chlamydia* and that some transcripts made in the absence of translation can be very stable (Ouellette et al., 2006).

## Dramatic Down-Trp selection of the joined chorismate/menaquinone pathway in *Chlamydiaceae*

In the chlamydiae the seven-step pathway to chorismate and the connecting menaquinone pathway can be considered to be a lengthy but simple, unbranched pathway. The *Chlamydiaceae* members differ from members of other *Chlamydiales* families in utilizing the DH6N variation rather than the classic DH2N pathway. In contrast, in organisms such as *E. coli* and *Streptomyces coelicolor*, the menaquinone pathway is but one of many connecting branches—resulting in a highly branched, complex system of biosynthesis. Figure 7 illustrates the Trp content of the enzymes of chorismate/menaquinone biosynthesis in *E. coli* (Ecol), *S. coelicolor* (Scoe), *C. trachomatis* (Ctra) as a representative of *Chlamydiaceae*, and in *P. amoebophila* (Pamo) as a representative of other *Chlamydiales*. Ecol and Pamo are similar in their utilization of the classic DH2N pathway (depicted with orange histogram bars in Figure 7), whereas Ctra and Scoe are similar in having the DH6N pathway routing (green bars). Extreme Down-Trp selection in Ctra (and all *Chlamydiaceae*) for enzymes of both the chorismate-pathway enzymes and the menaquinone pathway is apparent by examination of the summarized Trp content indicated by the horizontal bars at the bottom of Figure 7.

## Concluding perspective

The *Chlamydiaceae* exist in one of two alternative states. (i) A proliferative mode consists of two life-cycle phases: RBs are replicative bodies that exhibit high metabolic activity and are associated with acute disease; they parasitically exhaust the cellular resources and eventually cause lysis of the host cell in concert with the formation of EBs. The released EBs are infectious entities that find and infect new cell hosts. (ii) In the persistent mode, metabolic activity of the pathogen is greatly altered. Persistence is a survival mode that is proposed to be associated with environmental stress and subsequent survival in the absence of growth—a mode that may be reversed once the stress is relieved. It has been suggested that persistence, as defined by these terms, may be associated with a variety of chronic chlamydial infections (Gieffers et al., 2003; Seto et al., 2008; Ouellette et al., 2012), but this hypothesis requires vigorous *in vivo* validation (Byrne and Beatty, 2012).

There are surely unknown aspects of regulation in play, but at this time we can at least consider whether whole circuits of regulation might be activated or inactivated depending upon the Trp content of the regulator itself. An intriguing possibility is provided by Trip230, encoded by CT647 in *C. trachomatis*. It probably acts as a transcriptional activator and was suggested to function in folate metabolism (Lo et al., 2012). It has the highest p/P Trp ratio in the entire Ctra proteome. The menaquinone pathway of biosynthesis offers exciting research prospects because: (i) It is undoubtedly crucial for survival of either rapidly growing cells or cells in the persistent state of quiescence, and (ii) The menaquinone pathway of *Chlamydiaceae* is not present in the host or in typical beneficial flora, therefore providing multiple protein targets for new, specific antimicrobial agents.

The *Chlamydiales* are a group of highly specialized organisms evolved to survive in a unique environmental niche comprising a membrane-bound vacuole (inclusion) within the cytoplasm of the cells of the host. Survival within this niche requires that the chlamydiae be capable of sensing changes in host cell physiology that will evoke modulation of the chlamydial growth state (i.e., productive versus persistent growth). One key trigger for *Chlamydiales*, whether the host is a free-living amoeba or a human being, is the availability of a single metabolite. That metabolite is Trp. Since strong competition must exist for the limited amount of Trp available to the pathogen in the persistent mode, the protein assemblage supporting the persistent mode needs to compete advantageously under these conditions. Hence, evolutionary selection has occurred for Down-Trp proteins that characterize the persistent mode. In this light, the basis for such Down-Trp selection seems obvious. But why has Up-Trp selection occurred for proteins strongly engaged in the proliferative mode? We suggest that it is because this maximizes vulnerability to the completion of translation tasks whenever the shift to the persistent mode occurs. Lack of translational follow-through for the proliferative-mode set of proteins may not only facilitate the developmental transition but may also result in some proteolysis with release of small amounts of Trp then made available for the persistent-mode set.

### Conflict of interest statement

The authors declare that the research was conducted in the absence of any commercial or financial relationships that could be construed as a potential conflict of interest.
